# Differentiating Tuberculous and Pyogenic Spondylodiscitis: Part II: MRI/CT Features, Prediction Models, and the Role of PET/CT—A Narrative Review

**DOI:** 10.3390/diagnostics16162540

**Published:** 2026-08-12

**Authors:** Oana Maria Vanta, Anamaria Marian, Manuela Lenghel, Linda Ghib, Sonia Irina Vlaicu, Larisa Rotaru, Ileana Nicoară, Simona Rednic, Cristina Pamfil

**Affiliations:** 1 Department of Neurology, “Iuliu Hatieganu” University of Medicine and Pharmacy, Cluj Clinical County Emergency Hospital, 400012 Cluj-Napoca, Romania; 2 Department of Rheumatology, “Iuliu Hatieganu” University of Medicine and Pharmacy, 400012 Cluj-Napoca, Romania; anymaria.marian@gmail.com (A.M.); lindaghib@gmail.com (L.G.); 3 Department of Radiology, “Iuliu Hatieganu” University of Medicine and Pharmacy, 400012 Cluj-Napoca, Romania; lenghel.manuela@gmail.com; 4 Department of Internal Medicine, “Iuliu Hatieganu” University of Medicine and Pharmacy, Cluj Clinical County Emergency Hospital, 400012 Cluj-Napoca, Romania; vlaicus@yahoo.com; 5 Internal Medicine Department, Nicolae Testemiţanu, State University of Medicine and Pharmacy, 2004 Chișinău, Moldova; larisa.rotaru@usmf.md; 6 Department of Rheumatology, “Iuliu Hatieganu” University of Medicine and Pharmacy, Cluj Clinical County Emergency Hospital, 400012 Cluj-Napoca, Romania; ileana_nicoara@yahoo.com (I.N.); srednic.umfcluj@gmail.com (S.R.); cristinapamfil.umfcluj@gmail.com (C.P.)

**Keywords:** tuberculous spondylodiscitis, pyogenic spondylodiscitis, MRI, computed tomography, PET/CT, prediction models, spinal tuberculosis, vertebral osteomyelitis, imaging differentiation, diagnostic accuracy

## Abstract

Distinguishing tuberculous spondylodiscitis (TS) from pyogenic spondylodiscitis (PS) on imaging remains clinically important because the two entities differ in antimicrobial strategy, surgical timing, and the urgency of microbiological workup, yet no single imaging sign is pathognomonic for either diagnosis. When tissue confirmation is delayed, imaging becomes the primary tool that shifts pre-test probability and guides biopsy strategy—a role that is particularly critical in culture-negative disease, in antibiotic-exposed patients, and in settings with limited access to rapid mycobacterial diagnostics. This narrative review maps the imaging evidence most useful for routine TS-versus-PS differentiation, synthesizes recent prediction models and quantitative tools, and evaluates the adjunctive role of ^18^F-FDG PET/CT in biopsy-oriented decision-making. PubMed/MEDLINE was searched from inception to 31 March 2026 using pre-specified search terms across three concept blocks. This was a narrative review without formal pooling of quantitative estimates or prospective protocol registration. A secondary Scopus search identified no additional eligible records. Priority was given to comparative TS-versus-PS imaging cohorts, meta-analyses of MRI and CT features, prediction-model studies, and the recent radiomics and PET/CT literature; radiomics and deep-learning studies are appraised as a distinct evidence tier given their retrospective single-center derivation and predominantly internal-only validation. In total, 14 comparative TS-versus-PS imaging cohorts, three meta-analyses, and 14 prediction-model, quantitative-scoring, radiomics, or deep-learning studies formed the core evidence base. MRI yields the greatest differentiating information and is the recommended first-line modality. Features favoring TS include thoracic predominance, multilevel or non-contiguous involvement, relative early disc preservation, subligamentous spread, intraosseous abscesses, thin-walled paravertebral collections, and severe vertebral collapse with kyphotic deformity. Features favoring PS include lumbar predominance, early disc-endplate destruction, homogeneous inflammatory enhancement, facet-joint arthritis, and epidural phlegmon. CT adds osseous detail—including sequestra, subligamentous bone erosion, and paravertebral calcification—and remains the primary guidance modality for biopsy. Recent prediction models combining imaging and laboratory variables have shown high discriminative performance in derivation cohorts; however, most remain internally validated and locally calibrated. Pending external validation, none should be considered ready for unmodified clinical adoption, and they should therefore be used as probability modifiers rather than stand-alone diagnostic rules. ^18^F-FDG PET/CT is complementary rather than primary and is most useful when MRI is contraindicated or equivocal, in hardware-associated infection, and for whole-body staging in suspected disseminated tuberculosis. Non-infectious mimics and alternative infectious diagnoses should be considered when the imaging pattern is internally inconsistent or when standard cultures remain negative. Imaging findings should refine etiological probability and guide biopsy strategy, but should not replace tissue confirmation when tissue is feasible. Non-imaging evidence complementing this review is addressed in the companion manuscript, Part I.

## 1. Introduction

Tuberculous spondylodiscitis (TS) and pyogenic spondylodiscitis (PS) are the two principal infectious vertebral syndromes encountered in routine adult practice, yet they differ substantially in microbiological work-up, antimicrobial strategy, surgical timing, and the expected tempo of structural damage [1,2,3,4,5,6]. When tissue confirmation is delayed, imaging becomes the primary discriminator, shifting pre-test probability and guiding the urgency, trajectory, and microbiological priorities of biopsy [7,8,9,10]. That role is particularly important in culture-negative disease, in patients already exposed to antibiotics, and in settings where access to open biopsy or rapid mycobacterial diagnostics is limited [1,7,11].

The objective of this review is twofold: (1) to define the MRI and CT imaging phenotype that most reliably shifts pre-test probability toward TS or PS, and (2) to determine how prediction models, radiomics, and PET/CT can be incorporated into biopsy-oriented decision-making.

MRI remains the central modality for suspected native spondylodiscitis because it provides high anatomical resolution and depicts marrow edema, disc involvement, epidural disease, and paraspinal extension [7,8,9,12]. However, etiological differentiation rarely depends on a single sign [13,14]. In practice, the most useful interpretation is pattern-based and probabilistic, integrating vertebral level, number of involved segments, behavior of the disc-endplate complex, morphology of paraspinal and epidural collections, ligamentous spread, degree of osseous collapse, and CT-specific clues such as sequestra or calcification [7,12,14,15,16,17,18,19,20].

For clinicians managing back pain in medically complex adults, this distinction is especially consequential in older patients, those with diabetes or chronic kidney disease, after prior spinal procedures, and in immunocompromised hosts receiving glucocorticoids, biologics, chemotherapy, or transplantation-related immunosuppression [21,22,23,24,25,26,27,28]. In these settings, tuberculosis may present subacutely with attenuated inflammatory markers, whereas partially treated pyogenic disease may lose its usual acute phenotype [2,29,30,31]. This article constitutes Part II of a two-part narrative review: Part I addresses epidemiology, clinical features, laboratory markers, and tissue-based diagnosis, whereas the present review focuses on MRI/CT patterns, imaging-based prediction models, and the adjunctive role of PET/CT in biopsy-oriented decision making. Accordingly, this review is not intended merely to catalogue imaging signs; rather, it aims to connect specific MRI, CT, and PET/CT findings with biopsy targeting, prioritization of microbiological and mycobacterial testing, and selection of complementary imaging studies, so that imaging interpretation translates directly into diagnostic and biopsy-related action.

## 2. Methods

This manuscript is Part II of a two-part narrative review examining the differential diagnosis of tuberculous spondylodiscitis (TS) and pyogenic spondylodiscitis (PS). The narrative format was chosen because the comparative imaging literature is heterogeneous in study design, MRI protocols, case definitions, and endemic settings, and because the primary clinical objective—mapping the imaging evidence most useful for routine differentiation—is better served by structured synthesis than by pooled quantitative estimation. Non-imaging evidence (epidemiology, clinical features, laboratory markers, tissue acquisition, histopathology, and molecular diagnostics) is addressed in Part I and was not included in the search for this manuscript.

Searches of PubMed/MEDLINE were conducted from database inception to 31 March 2026 using combinations of the following terms across three concept blocks, combined as Block 1 AND (Block 2 OR Block 3): (1) condition—“tuberculous spondylitis”, “spinal tuberculosis”, “tuberculous spondylodiscitis”, “pyogenic spondylodiscitis”, “pyogenic spondylitis”, “vertebral osteomyelitis”, “spondylodiscitis”; (2) modality—“MRI”, “magnetic resonance imaging”, “CT”, “computed tomography”, “PET/CT”, “whole-spine MRI”, “nuclear medicine”, “SPECT/CT”, “FDG”, “^18^F-FDG”; and (3) evidence type—“prediction model”, “radiomics”, “deep learning”, “diagnostic accuracy”, “imaging features”, “abscess”, “sequestra”, “subligamentous spread”. A secondary search of Scopus was performed using identical terms and date range; no additional eligible records were identified. Reference lists of included comparative studies, meta-analyses, and five high-citation imaging-focused narrative reviews published after 2015 were systematically hand-searched [7,8,9,10,11] by two co-authors to identify additional relevant records; these sources are listed in Supplementary File S1, which also provides the full reproducible Boolean search strings and the corresponding MeSH controlled-vocabulary terms. No protocol was prospectively registered, consistent with the narrative review design; the review question and eligibility criteria were defined prior to searching. Title and abstract screening and full-text eligibility assessment were performed by the two authors (L.G. and A.M.). Eligibility decisions were reviewed for consistency on a 20% random sample of screened records by a co-author (C.P.), with discordant decisions resolved by discussion. Formal dual independent screening of all records was not performed, which is acknowledged as a limitation of the narrative review design. Because this article was designed as a narrative review rather than a systematic review, the literature search was conducted iteratively and by topic to support the manuscript’s predefined imaging domains. Formal de-duplicated title and abstract screening of all records retrieved by broad database searches was not performed; accordingly, the article is not presented as compliant with the Preferred Reporting Items for Systematic Reviews and Meta-Analyses (PRISMA) guidelines. To improve transparency and reproducibility, the Methods Section has been expanded, and the full electronic search strategies are provided in Supplementary File S1.

Records were considered eligible if they reported MRI, CT, or PET/CT findings in patients with confirmed or probable spondylodiscitis, or if they directly compared imaging features, diagnostic scores, or nuclear medicine findings between TS and PS on at least one parameter. For the purposes of eligibility assessment, confirmed spondylodiscitis was defined as microbiologically established disease by a positive culture or a validated molecular test from spinal or paraspinal tissue or blood; probable spondylodiscitis was defined as a clinical and radiological diagnosis meeting the Infectious Diseases Society of America (IDSA) 2015 [32] or Société de Pathologie Infectieuse de Langue Française (SPILF) 2022 [33] guideline criteria in the absence of microbiological confirmation. Prediction models and radiomics studies were included if they reported internal or external validation performance. Imaging-only studies restricted to postoperative, hardware-associated, or pediatric-only populations were used to inform the corresponding dedicated subsections rather than the main comparative synthesis. Case reports and series of fewer than 10 patients were excluded unless they documented imaging findings not available in larger series.

Studies were judged clinically representative when they used consecutive or unselected enrolment, applied a microbiological or histopathological reference standard, and reported findings from populations with TB burden representative of routine referral practice; studies meeting fewer criteria were retained only when they reported findings not otherwise available. Priority was given to comparative TS-versus-PS imaging cohorts, meta-analyses of MRI or CT features, prediction-model studies, and recent radiomics and PET/CT literature. Radiomics and deep-learning studies are appraised as a distinct evidence tier from established imaging sign literature, given their retrospective single-center design and internal-only validation in most cases. Formal risk-of-bias instruments (e.g., QUADAS-2) were not used because this is a narrative rather than a systematic review; instead, key studies are characterized in Table 1 and Table 2 in the sections below by design, reference standard, and population representativeness, with criteria applied consistently to comparative and prediction-model studies alike. Instead, studies are characterized throughout the Results according to three dimensions: study design (prospective vs. retrospective; consecutive vs. selected enrolment), reference standard (microbiologically confirmed vs. clinically probable), and population representativeness (single-center tertiary vs. multicentre or population-based). This qualification is applied where it materially affects the interpretation of reported estimates. Quantitative estimates of diagnostic performance are presented as reported in primary studies and are not pooled; where studies report discordant results, the range of estimates is stated with an explanation of likely sources of discordance. Pediatric imaging data are used to characterize age-specific phenotypic differences rather than to construct a separate diagnostic algorithm.

Meta-analyses and multicenter comparative cohorts were weighted most heavily, followed by pathogen-confirmed single-center cohorts; prediction-model, radiomics, and deep-learning studies were treated as hypothesis-generating and did not override conclusions from comparative imaging cohorts.

The final reference set included 97 records selected for their relevance to the imaging-based differentiation between TS and PS. To provide a numerical overview of the literature base, records were grouped according to their primary role in the narrative synthesis: reviews, guidelines, and general background/epidemiology sources (*n* = 27); comparative TS-versus-PS MRI/CT imaging studies (*n* = 24); PET/CT and nuclear medicine studies (*n* = 14); prediction-model, radiomics, and deep-learning studies (*n* = 11); CT-specific studies (*n* = 7); pediatric spinal infection studies (*n* = 6); mimics and differential-diagnosis sources (*n* = 6); and whole-spine imaging studies (*n* = 2). Categories were assigned based on each reference’s primary role in the manuscript, recognizing that some records overlap thematically.

## 3. Anatomical and Pathobiological Basis of Imaging Differences

Some anatomical determinants are well established, notably age-related regression of intervertebral disc vascularity; others, particularly the proposed role of Batson’s plexus in the distribution of tuberculous spondylodiscitis (TS), remain inferential, extrapolated from anatomical studies rather than directly tested against imaging outcomes [1,2,3,4,5,6]. Spinal infection usually arises through hematogenous dissemination from a distant focus, with both arterial and venous routes contributing to its distribution [1,2,3,4,5,6]. Its imaging phenotype reflects the interaction between vertebral vascular anatomy and pathogen-specific biology [1,7,34].

Age-related changes in vertebral and discal vascularity influence the imaging pattern of spinal infection [1,7,34]. In children, persistent direct disc vascularity permits primary infection and a disc-centered pattern [35,36]. In adults, regression of this supply leaves the disc relatively avascular, so infection usually begins in the adjacent endplate and subchondral marrow before extending into the disc [1,34]. This explains why primary discitis is more common in children, whereas adult disc involvement is usually secondary to adjacent vertebral infection [1,34,35,36,37].

**Figure 1:**
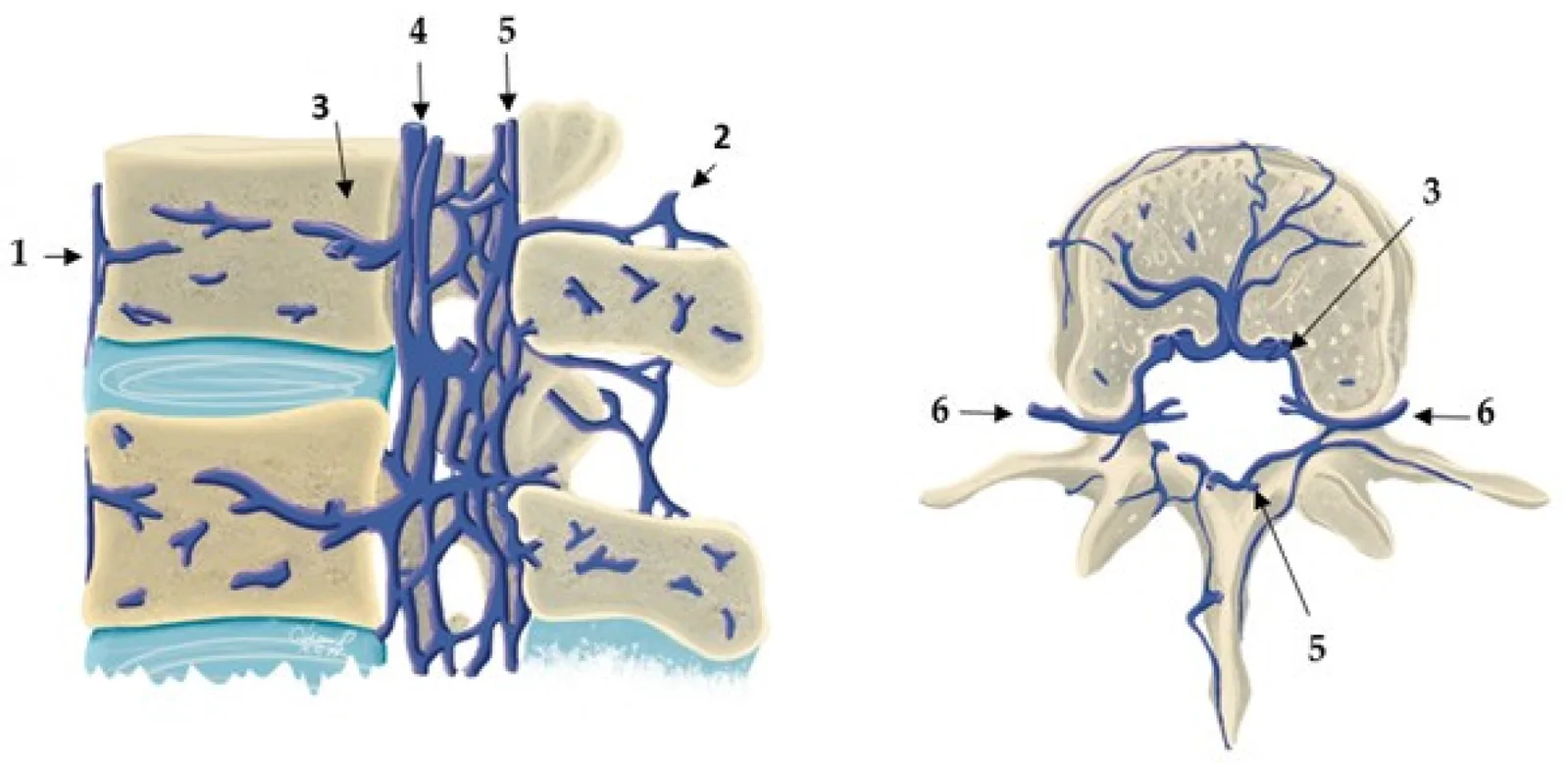
Batson’s valveless vertebral venous plexus. Sagittal and axial schematic views of the vertebral venous system, including the segmental venous connection (1), posterior venous branch (2), basivertebral venous channels/vein (3), anterior internal vertebral venous plexus (4), posterior/internal vertebral venous plexus (5), and intervertebral veins (6). Its valveless architecture permits bidirectional flow and provides a traditional anatomical framework for vertebral seeding and occasional non-contiguous spread in TS, although this role remains inferential rather than directly validated against imaging outcomes.

These anatomical principles are particularly relevant to the adult pattern of tuberculous spondylodiscitis (TS), which classically involves the anterior vertebral body [4,29]. This distribution is best understood as the result of hematogenous seeding shaped by vertebral vascular anatomy, while Batson’s valveless paravertebral venous plexus is traditionally invoked as an additional pathway that may facilitate vertebral seeding from distant foci and help explain multilevel or non-contiguous disease [1,3,4,29]. Its specific contribution, however, is anatomically plausible rather than directly demonstrated [3,29]. Anterior infection may extend beneath the anterior longitudinal ligament, producing subligamentous spread, multilevel paradiscal involvement, and large paraspinal collections [1,3,7,29]. By contrast, pyogenic spondylodiscitis (PS) more often follows an endplate-disc suppurative pattern, with earlier disc destruction and less orderly longitudinal extension [1,2]. A schematic overview of Batson’s valveless vertebral venous plexus is shown in Figure 1.

Microbiological behavior and host response further shape the imaging phenotype [1,6]. The granulomatous, caseating, and relatively indolent response to *Mycobacterium tuberculosis* is consistent with progressive vertebral destruction, subligamentous extension, thin-walled “cold” abscesses, collapse, and extensive soft-tissue spread despite modest systemic inflammation [3,29,30,38]. In contrast, pyogenic organisms—most commonly *Staphylococcus aureus*—induce an acute neutrophilic suppurative response associated with earlier endplate damage, faster disc-space involvement, and a greater tendency toward epidural phlegmon or facet joint septic arthritis rather than smooth, well-defined abscess formation [1,2,17,39]. However, these associations are probabilistic, and no individual imaging feature is sufficiently specific to establish the etiology in isolation [14,15].

Understanding these determinants helps explain why tuberculous and pyogenic infection often differ in their distribution, pattern of vertebral and disc involvement, abscess morphology, and longitudinal extent on MRI and CT. Together, these concepts provide a framework for interpreting the imaging patterns discussed below.

## 4. Conventional Radiography

Plain radiography has limited value for early etiological separation, but it is not obsolete [6,7,12]. Radiographic abnormalities usually emerge only after substantial endplate and vertebral damage has occurred; normal films do not argue against either diagnosis [2,7,12]. Once abnormal, radiographs may still provide useful context: aggressive disc-space narrowing early in the course is more compatible with PS, whereas anterior vertebral body loss, progressive angular kyphosis, gibbus deformity, large prevertebral shadows, and calcification within a paraspinal soft-tissue mass are classic late clues favoring TS [4,13,38,40]. Published estimates of sensitivity and specificity for direct TS-versus-PS differentiation by radiography are sparse, reflecting its limited discriminative role relative to MRI and CT; accordingly, radiography is best regarded as a baseline structural study rather than a discriminative test [7,32,33,40].

## 5. Computed Tomography

**Figure 2:**
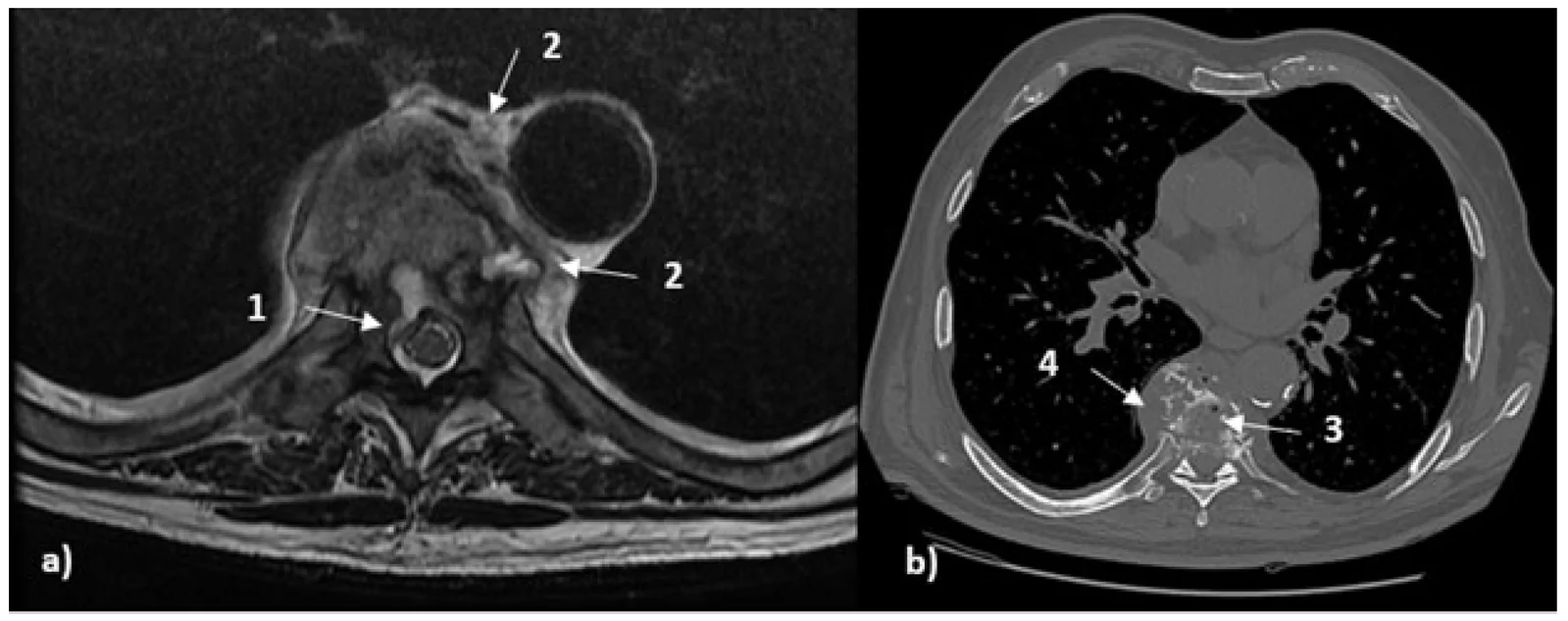
Tuberculous pattern on MRI and CT: body-centred destruction with thin-walled paravertebral extension. Axial T2-weighted MRI (**a**) and axial CT (**b**) at D8 show heterogeneous vertebral body involvement, epidural abscess with dural indentation (1), and sharply marginated paravertebral collections (2, 4) with associated osseous destruction (3)—an imaging constellation that shifts probability towards TS rather than ordinary PS.

**Figure 3:**
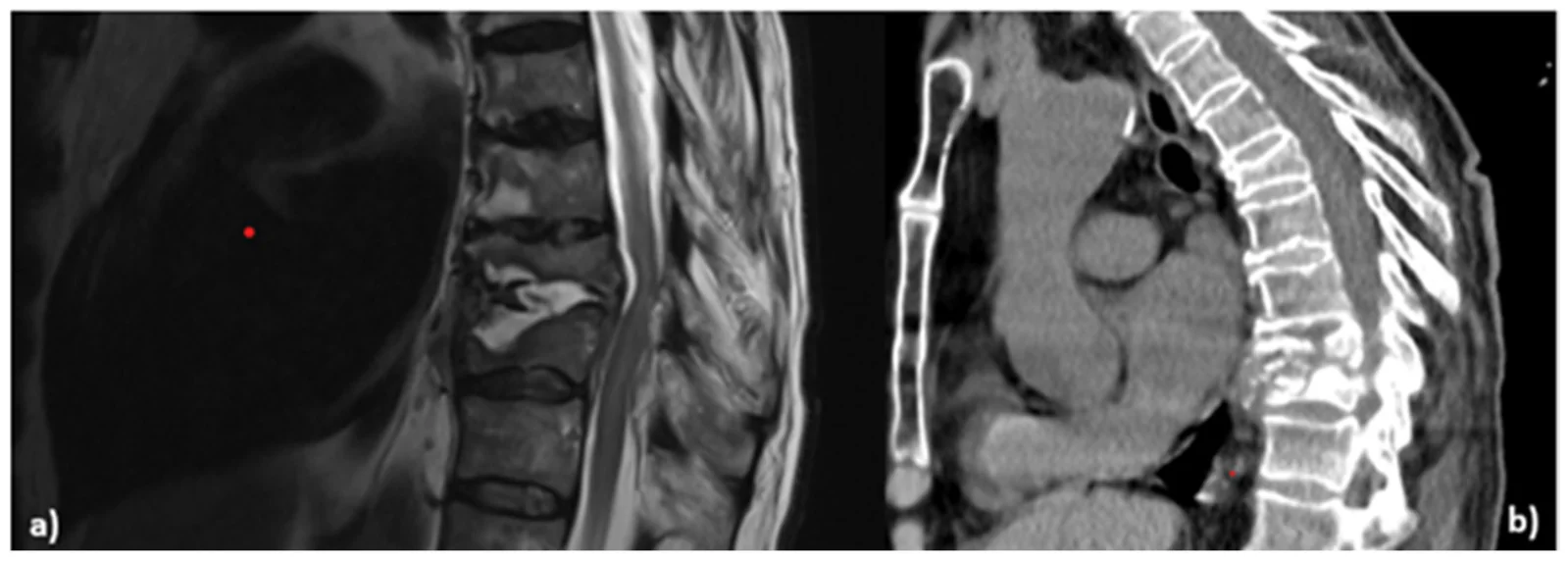
Pyogenic spondylodiscitis on MRI and CT. Sagittal T2-weighted MRI (**a**) and sagittal CT (**b**) demonstrate T9–T10-centered destruction of the disc–endplate complex, with marked disc-space involvement, irregular erosion of the opposing endplates, and ill-defined paravertebral and epidural inflammatory extension causing spinal canal narrowing and cord compression. The disc-centered destructive pattern and phlegmonous soft-tissue extension favor PS over TS. Vertebral collapse alone is not discriminatory, as it may also occur in advanced TS.

CT should be viewed as complementary rather than secondary to MRI [7,12]. In the TS-versus-PS differential, CT contributes information that MRI may depict less clearly, including cortical breach, sequestra, marginal sclerosis, paravertebral calcification, deformity geometry, and safe biopsy access [40,41]. It is therefore particularly helpful when MRI suggests infection but the osseous pattern remains uncertain, and it remains the main guidance modality for image-guided tissue sampling [40,41,42,43]. Representative tuberculous and pyogenic MRI/CT appearances are shown in Figure 2 and Figure 3.

When CT is informative, findings that tend to raise suspicion for TS include a destructive process centered in the vertebral body, subligamentous bone erosion, relatively preserved disc morphology early in the disease, larger or ‘massive’ sequestra, paraosseous bone formation extending over a longer craniocaudal segment, osteosclerotic or osteoporotic margins rather than sharply aggressive endplate lysis, and more pronounced kyphotic angulation [41,44,45,46,47,48,49]. Paravertebral calcification is uncommon but, when present, remains a useful clue to TS [44,48,50]. By contrast, PS more often shows dominant endplate destruction with rapid disc-height loss, shorter-segment cortical erosion, less organized bone formation, and less marked angular deformity [2,40,41]. These CT findings are stage-dependent and probabilistic: many become more readily detectable as structural damage progresses, and their sensitivity is limited early in the disease course [7,12,40]. Consequently, a normal or minimally abnormal CT does not exclude either TS or PS [7,40].

These observations have been incorporated into diagnostic models. Liu et al. developed a CT-based model using bone destruction pattern, distribution of bone formation, sequestra, residual vertebral shape, kyphosis, and vertebral overlap [41]. Developed in a single-center retrospective cohort and evaluated using an internal derivation–validation split, the model achieved a sensitivity of 85.6%, specificity of 87.8%, and an AUC of 0.95; no external validation was reported in the original study [41]. A more recent pathogen-confirmed model by Zhang et al. integrated CT, MRI, and laboratory data and identified four especially informative variables: white blood cell count < 7.265 × 10^9^/L, skip lesions or involvement of three or more segments, massive sequestra formation, and subligamentous bone destruction [51]. The derivation model yielded 91.4% sensitivity, 95.7% specificity, and an AUC of 0.981; the authors also reported similar performance after internal bootstrap validation and in an external validation set [51]. Despite these promising results, the high AUC values should be interpreted with caution: both models were developed retrospectively in relatively small, selected cohorts, and broader independent multicenter validation and calibration assessments are required before unmodified clinical adoption. Taken together, these data suggest that CT is most useful when the clinician is asking whether the infection behaves more like a slower body-centered granulomatous process or a rapidly destructive endplate-disc infection [7,12,41,44,49].

## 6. MRI Differentiators

MRI is the key imaging modality for etiological differentiation, although its contribution is best understood as probabilistic rather than definitive [14,16,18,40]. The recent meta-analysis by Chen et al., which pooled 32 studies, suggests that no single finding is pathognomonic, but several features are reported more often in TS, including subligamentous spread, thin and regular abscess walls, epidural extension, vertebral collapse and kyphotic deformity [15]. Disc signal abnormality and disc-height loss, by contrast, are more often encountered in PS [15]. Ligamentous and posterior-element signs can provide additional support, but they usually need to be interpreted in context [14,15,16]. For practical clinical reading, these MRI findings can be grouped into four overlapping domains: distribution and anatomical extent; disc-endplate behavior; vertebral destruction and marrow signal; and the pattern of paravertebral or epidural soft-tissue disease [14,15,18,20]. Formal inter-reader reliability data for most of these MRI signs are limited; reported reproducibility largely reflects single-center reader performance under non-standardized protocols rather than externally validated agreement across institutions and MRI sequences. Table 1 summarises the imaging features that most consistently favour TS or PS with key interpretive caveats.

**Table 1:** Imaging features favoring tuberculous or pyogenic spondylodiscitis.

Domain	Findings Favoring TS	Findings Favoring PS
Predominant level and extent ^a^	Thoracic predominance; Multilevel disease (≥2 vertebral bodies); Non-contiguous skip lesions	Lumbar predominance; Usually contiguous single-level disease
Disc-endplate complex ^b^	Relative early disc preservation; Vertebral destruction disproportionate to disc injury	Early disc signal change; Rapid disc-space loss; Marked endplate destruction
Vertebral body signal ^c^	Heterogeneous T1/T2-STIR signal and inhomogeneous enhancement; Intraosseous abscesses more common	More homogeneous marrow and soft-tissue enhancement
Pattern of osseous destruction and alignment ^d^	Severe body-centered destruction; Collapse > 50%; Vertebral overlap and kyphotic deformity	Moderate destruction < 50%; Usually less angular deformity initially
Paravertebral collection/epidural disease ^e^	Large, thin-walled, well-defined abscesses; Epidural abscess	Thicker or more irregular walls; Ill-defined inflammatory tissue or phlegmon more typical; Epidural phlegmon
Ligamentous spread ^f^	Subligamentous spread beneath the anterior longitudinal ligament	Less prominent subligamentous spread; Infection is more often centered at the disc-endplate interface
Anterior meningovertebral ligament ^g^	Preserved in the setting of anterior epidural abscess	More often involved
Facet and posterior elements ^h^	Posterior element involvement may support TS	Facet-joint arthritis more often favors PS
CT-specific clues ^i^	Massive sequestra; Marginal sclerosis; Paravertebral calcification	Rapid endplate erosion with disc collapse
PET/CT contribution ^j^	May reveal multifocal or disseminated TB; Higher SUV can occur, but is **not** specific.	Useful in early disease, hardware-associated infection, and metastatic bacterial foci

TS: tuberculous spondylodiscitis; PS: pyogenic spondylodiscitis; STIR: short tau inversion recovery; MRI: magnetic resonance imaging; PET/CT: positron emission tomography–computed tomography; SUV: standardized uptake value. ^a^ Whole-spine survey warranted when TS is suspected; absence of skip lesions does not exclude TS. ^b^ Advanced TS and antibiotic-exposed PS may overlap. ^c^ Signal pattern alone is not specific. ^d^ Advanced, untreated PS can still collapse severely. ^e^ Well-defined abscesses can occasionally occur in indolent bacterial infection. ^f^ One of the most reproducible MRI clues favoring TS. ^g^ Helpful but specialized sign; use as supportive rather than stand-alone evidence. ^h^ Neither finding should be interpreted in isolation. ^i^ Paravertebral calcification is highly suggestive, but uncommon. ^j^ PET/CT refines extent, not etiology. Features summarised from references cited in the main text.

### 6.1. Distribution and Anatomical Extent

TS shows a consistent thoracic predominance, whereas PS is more often lumbar [14,17,39]. Multilevel disease, involvement of three or more vertebral bodies and, especially, non-contiguous skip lesions all increase the probability of TS, particularly when disease extends above and below an apparently less involved level [14,15,30]. In the multicenter MRI study by Wang et al., skip lesions occurred in 28.6% of TS cases and in none of the PS cases; involvement of three or more vertebral [15] bodies was likewise much more frequent in TS [52]. In practice, the clinician should not assume that the symptomatic spinal level is the only level involved [6,7,44]. Current evidence supports whole-spine assessment in suspected disco-vertebral infection, a strategy that is particularly relevant in TS because clinically silent non-contiguous lesions may alter staging, biopsy targeting, and surgical planning [14,32,53,54].

Subligamentous spread is among the more reproducible MRI features favoring TS across both classic studies and recent meta-analysis [14,15,16,55]. Enhancement or lifting of the anterior longitudinal ligament by a long-segment collection is particularly helpful when it coexists with relative disc preservation [14,15,17]. A smaller AJNR series also suggested that preservation of the anterior meningovertebral ligament in cases with anterior epidural abscess may favor TS; in that study, sensitivity was 83.3% and specificity was 100% [56]. Posterior element involvement may support TS, whereas facet-joint arthritis favors PS, although neither finding should be interpreted in isolation [10,15,57].

### 6.2. Disc-Endplate Behavior

PS often begins at the vascularised endplate-disc junction and therefore tends to produce early disc T2 hyperintensity, loss of disc height, endplate blurring, and relatively homogeneous inflammatory change across the discovertebral unit [2,14,16,38]. TS more often begins in the anterior vertebral body and reaches the disc secondarily; accordingly, relatively early disc preservation, particularly when it seems disproportionate to the degree of vertebral destruction, remains a helpful clue favoring TS [3,14,39,44]. This distinction is most informative in untreated early or intermediate disease [15,58,59]. It becomes less secure in advanced TS, in older adults with marked degeneration, or after antimicrobial pretreatment, when disc destruction can no longer be interpreted as uniquely pyogenic [14,15,58,59].

### 6.3. Vertebral Destruction and Marrow Signal

Compared with PS, TS more often shows heterogeneous T1 hypointensity, heterogeneous T2/STIR hyperintensity and inhomogeneous gadolinium enhancement rather than a uniform marrow signal pattern [14,55,60]. The vertebral lesion is also often more severe, more body-centered, and more likely to contain intraosseous abscesses with rim enhancement [14,55]. Severe body destruction or loss of more than 50% of vertebral height, vertebra plana-like collapse, and kyphotic angulation all shift probability towards TS, although overlap with advanced PS is well recognized [15,29,61]. The 2026 propensity score-matched analysis by Bulut et al. is particularly useful because it re-examined classic signs after balancing baseline differences between groups: thoracic location and vertebral destruction >50% remained associated with TS, whereas epidural phlegmon favored PS [61].

### 6.4. Paravertebral and Epidural Disease

**Figure 4:**
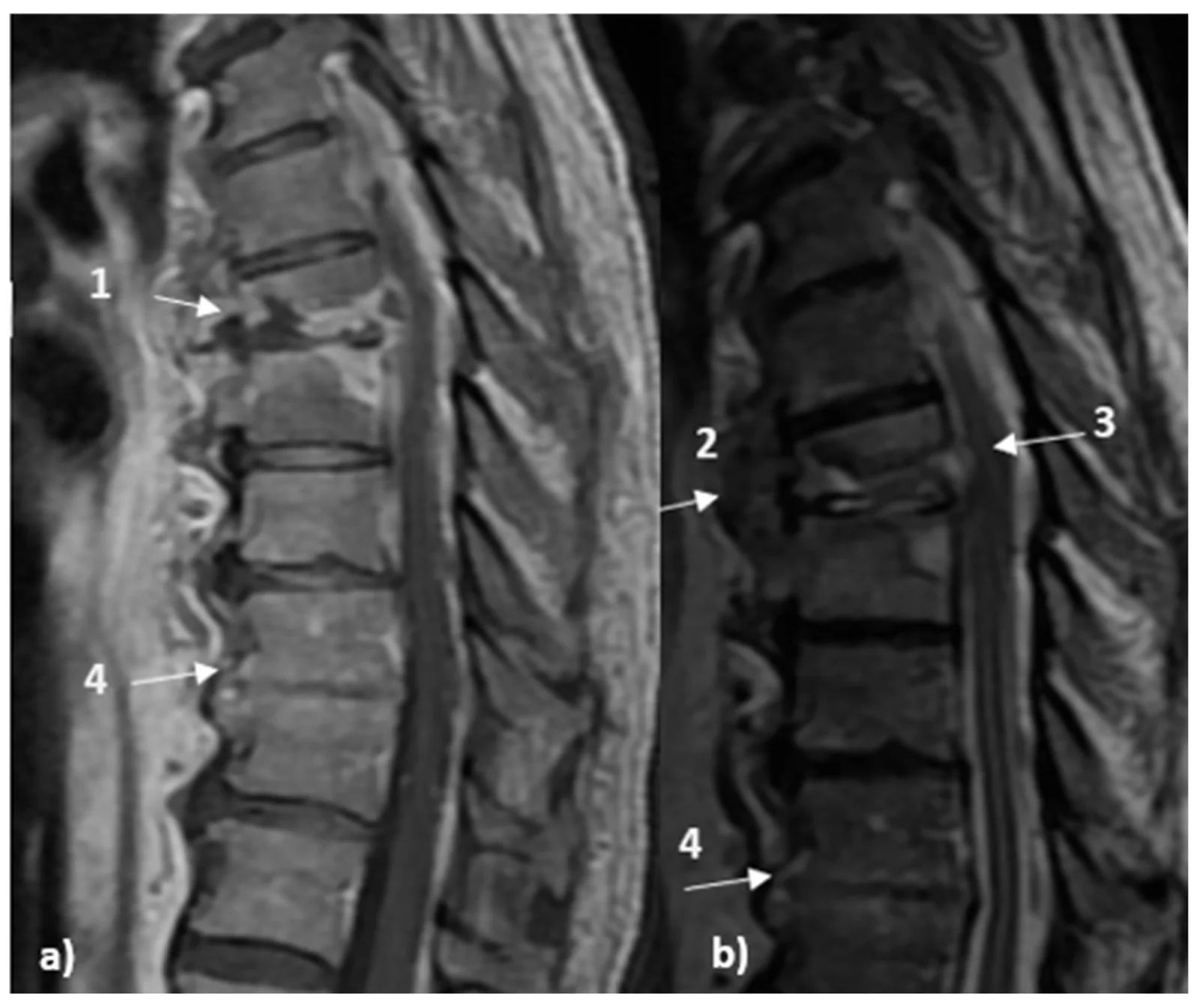
Tuberculous pattern with non-contiguous disease: skip lesions and multilevel extension. Sagittal post-contrast T1-weighted (**a**) and T2-weighted (**b**) images show T7–T8 destruction with anterior collapse (1), heterogeneous enhancement, paravertebral (2) and epidural abscesses (3), and a second non-contiguous level at T10–T11 (4), a distribution that favors TS.

The paravertebral and epidural compartments are often among the most informative areas for differentiation [7,10,15]. TS classically produces large paravertebral collections with thin, smooth walls and sharply defined post-contrast margins, frequently extending beneath the anterior longitudinal ligament across multiple levels [4,14,55,60]. Epidural abscess is common in TS, but the epidural component often retains the appearance of a true abscess rather than an ill-defined inflammatory phlegmon [14,15,55,62]. By contrast, PS is more often associated with thick or irregular abscess walls, poorly marginated paravertebral inflammatory tissue, facet-joint arthritis, and epidural phlegmon [14,39,55,61,62]. Tanaviriyachai et al. incorporated these features into a seven-item MRI score in which thoracic location, absence of epidural phlegmon, subligamentous spread, intraosseous abscess, well-defined paravertebral abscess, epidural abscess, and absence of facet-joint arthritis independently predicted TS; a score ≥14/29 yielded 97.6% sensitivity and 92.5% specificity [62]. Because definitions of ‘epidural abscess’ and ‘phlegmon’ vary across institutions and studies, comparability of reported diagnostic performance is limited, and standardization of these terms is needed. A representative tuberculous pattern with collapse, skip disease, and epidural/paravertebral abscesses is shown in Figure 4.

### 6.5. Pitfalls and Overlap

The findings summarized above are probabilistic tendencies derived from group-level comparisons, not deterministic rules applicable to individual patients; overlap is expected, and increases in the clinical scenarios discussed below. Imaging overlap is most evident in advanced untreated PS, late TS with secondary disc collapse, partially treated infection, and postoperative or hardware-associated disease [11,20,58,59]. In these settings, interpretation is often more reliable when based on the overall constellation than on any single sign: does the process look predominantly body-centered, subligamentous and abscess-forming, or predominantly discovertebral and phlegmonous? [15,20,40]. Radiologists should also be cautious not to overcall TS solely on the basis of paravertebral soft-tissue findings, because well-defined collections can occasionally occur in indolent bacterial infection [57,63,64,65]. Conversely, the absence of skip lesions does not exclude TS; most TB cases remain contiguous [15,52]. The role of functional MRI techniques in this differential diagnosis remains investigational [66]. Small DCE-MRI studies suggest that stronger disc enhancement and different time-intensity kinetics may favour PS, but these methods have not entered routine practice [66].

## 7. Prediction Models and Emerging Radiomics

### 7.1. Clinically Oriented Scoring Systems

Recent prediction models aim to make imaging interpretation more reproducible, but they should be viewed as aids to clinical reasoning rather than autonomous classifiers [6,41,50,51,52,61,62,67]. The most clinically usable models are not purely radiomic; instead, they combine a limited number of imaging signs with readily available laboratory variables [41,50,51,61,67]. Across studies, recurrent predictors in TS include skip lesions, subligamentous spread, abscess morphology, sequestra, vertebral destruction, and a relatively low inflammatory burden [41,50,51,52,61,62,67]. The PLoS One model by Lertudomphonwanit et al. integrated paraspinal abscess, white cell count ≤9700/mm^3^, neutrophil fraction ≤78%, and ESR ≤92 mm/h, achieving an AUC of 0.921 for TS; however, it was derived from a single-center cohort retrospectively and has not been externally validated [67]. The 2023 retrospective case–control model by Liu et al. combined laboratory, CT, and MRI variables and again highlighted low inflammatory burden, skip lesions, sequestra, and subligamentous spread [50]. These models are clinically attractive because they reflect the way clinicians already reason—by combining imaging patterns with clinical and laboratory data, rather than interpreting MRI in isolation—but their performance must be interpreted in relation to the reference standard, validation design, and clinical setting.

### 7.2. Radiomics and Machine Learning

Radiomics and deep-learning approaches are increasingly represented in the literature, but for now, they remain adjunctive [68]. A CT-based clinical-radiomics nomogram showed AUCs of 0.891 in the training cohort, 0.830 in the internal validation cohort, and 0.877 in a separate test cohort, with calibration curves reported for the development cohorts [69]. More recently, a contrast-enhanced MRI deep-learning radiomics nomogram by Yang et al. achieved excellent discrimination in the development cohort but lower performance on external testing, suggesting both the potential to detect textural differences beyond conventional visual assessment and some degree of overfitting to the development dataset [70]. A 2026 two-center study by Chen et al. proposed simpler MRI signal-based tools—the epidural abscess signal (EAS) and vertebral body signal (VBS) scores—with derivation AUCs of 0.888 and 0.870, respectively, followed by prospective validation in a small second cohort [71]. Although encouraging, the limited size of the validation cohort restricts conclusions regarding generalizability [71]. These radiomics and deep learning models were generally developed in modest retrospective cohorts, often with fewer than 200 patients, variable TS/PS class balance, and limited independent validation, raising concerns about overfitting [69,70,72,73,74]. Robustness to class imbalance, scanner and protocol variation, feature-selection choices, and calibration has not been consistently reported [68,69,70,71,72,73,74]. Most models also remain geographically localized to TB-endemic settings, limiting their transportability to lower-prevalence populations and underscoring the need for larger, independent multicenter validation before routine clinical use [69,70,71,72,73,74].

### 7.3. How These Models Should Be Used

At present, imaging scores are more appropriately treated as probability modifiers than as stand-alone treatment rules [6,68]. Their calibration is likely to shift with local TB prevalence, referral case-mix, MRI protocols, reader expertise, and biopsy practice, so a score derived in a high-endemic referral cohort may overestimate TS when applied in a low-prevalence setting [69,72,73,74]. Current models may help prioritize whole-spine imaging, biopsy strategy, or the breadth of microbiological testing, but they do not justify empirical anti-tuberculous therapy or omission of biopsy solely on the basis of an imaging score [69,70,73]. None should be considered ready for unmodified clinical adoption until broader external validation and local calibration are in place. Selected models and emerging AI tools are summarized in Table 2.

**Table 2:** Selected diagnostic models and scores (to be used as probability modifiers pending external calibration).

Study	Input	Dominant Predictors	Performance and Caveats	Validation Status and Calibration
Liu et al. 2021 [41]	CT	Bone destruction pattern, paraosseous bone formation, sequestra, residual vertebral shape, kyphosis, vertebral overlap	Sensitivity 85.6%; specificity 87.8%; AUC 0.95. Strongly CT-centric.	Internal split-sample validation; calibration not reported; no external validation.
Lertudomphonwanit et al. 2023 [67]	Clinical + laboratory + imaging	Paraspinal abscess, WBC ≤ 9700/mm^3^, neutrophils ≤ 78%, ESR ≤ 92 mm/h	AUC 0.921. Clinically interpretable, but incorporates non-imaging biomarkers.	Derivation cohort only; Hosmer–Lemeshow model-fit test reported. No external validation.
Tanaviriyachai et al. 2024 [62]	MRI score	Thoracic lesion, no epidural phlegmon, subligamentous spread, intraosseous abscess, well-defined paravertebral abscess, epidural abscess, no facet arthritis	Cut-off ≥ 14/29: sensitivity 97.6%; specificity 92.5%; AUC 0.96. Practical and interpretable.	Internal bootstrap validation; calibration plot and Hosmer–Lemeshow test reported. No external validation.
Zhang et al. 2024 [51]	CT + MRI + WBC	WBC < 7.265 × 10^9^/L, skip lesion or ≥3 segments, massive sequestra, subligamentous bone destruction	Sensitivity 91.4%; specificity 95.7%; AUC 0.981. Pathogen-confirmed cohort.	Internal bootstrap and external validation reported; calibration not reported.
Wu et al. 2022 [69]	CT clinical-radiomics nomogram	CT-derived radiomics score combined with clinical risk factors	AUC 0.891 in training, 0.830 in internal validation, and 0.877 in the test cohort. Investigational.	Internal split-sample validation and additional same-center test cohort; calibration curves and Hosmer–Lemeshow tests reported; no independent external validation Geographic external validity remains limited.
Wang et al. 2024 [52]	MRI nomogram	Non-contiguous multivertebral disease, severe bone lesions, vertebral intraosseous, epidural and paraspinal abscesses among selected predictors	Selected cut-off: sensitivity 95.2%; specificity 94.8%.	Internal bootstrap validation within a multicenter retrospective cohort; calibration curve reported; no independent external validation.
Yang et al. 2025 [70]	CE-MRI deep learning radiomics	Contrast-enhanced image texture plus selected clinical features	AUC 0.994 in training and 0.859 on external testing. Workflow complexity limits routine adoption.	External-center testing; calibration curves reported. The external cohort was small.
Chen et al. 2026 [71]	MRI quantitative signal scores	EAS (epidural abscess signal) and VBS (vertebral body signal) thresholds	Derivation AUCs: EAS 0.888 and VBS 0.870. Simpler than full radiomics.	Prospective second-center validation after retrospective derivation; the validation cohort was small. Formal calibration analysis not reported.

AUC: area under the receiver operating characteristic curve; CE-MRI: contrast-enhanced magnetic resonance imaging; CT: computed tomography; EAS: epidural abscess signal; ESR: erythrocyte sedimentation rate; MRI: magnetic resonance imaging; VBS: vertebral body signal; WBC: white blood cell count. Most published models were developed retrospectively in geographically localized cohorts, and formal calibration was reported inconsistently. Internal validation was defined as split-sample, cross-validation, or bootstrap assessment within the source dataset. External validation was defined as evaluation in an independent cohort not used for model development; same-center holdout samples were classified as internal validation. Calibration was considered reported only when a calibration plot, goodness-of-fit test, calibration slope or intercept, or another quantitative calibration metric was provided.

## 8. When the Imaging Differential Is Not Binary

### 8.1. Alternative Infectious Diagnoses

The TS-versus-PS framework is clinically useful, but it does not exhaust the differential diagnosis. Fungal spondylodiscitis, brucellar disease, melioidosis and actinomycotic infection can reproduce combinations of vertebral-body destruction, relative disc preservation or large paraspinal collections that resemble TS; conversely, indolent or partially treated bacterial infection may simulate TB [57,63,64,75,76]. Recent broad-pathogen reviews emphasize that these alternatives become more plausible when the host is immunocompromised, the epidemiological context is distinctive, standard cultures remain negative, or the imaging pattern is internally inconsistent with ordinary PS [10,57,58,59].

### 8.2. Important Non-Infectious Mimics

Non-infectious mimics should also be considered when MRI is interpreted in isolation. Modic type 1 degeneration, acute Schmorl node, dialysis-related or neuropathic spondyloarthropathy, postoperative granulation tissue, SAPHO/Andersson lesions, and malignant infiltration can all produce endplate edema and enhancement [77,78,79,80]. Features that may pull interpretation away from infection include a vacuum disc, absence of paraspinal or epidural inflammatory extension, a dominant posterior-element tumor mass, exuberant sclerosis or ossification, and marked discordance between imaging severity and the inflammatory phenotype [77,78,79]. In equivocal cases, CT, diffusion-weighted MRI, interval imaging, and—above all—tissue diagnosis remain decisive [6,9,40,58], as summarized in Table 3.

### 8.3. Pediatric Considerations

Pediatric data are retained because vertebral and disc vascular anatomy alters the imaging phenotype enough to make simple adult extrapolation unsafe [36,81]. In children, persistent disc vascularity makes pyogenic disease more disc-centered, with earlier disc T2 hyperintensity and narrowing; pediatric TS remains more vertebral-body-dominant, with multilevel destruction, subligamentous collections, and more rapid deformity progression [35,36,37,81,82,83]. Whole-spine MRI is therefore particularly important when TB is suspected, but adult TS-versus-PS heuristics should be applied with caution in children [36,83].

## 9. PET/CT and Other Nuclear Medicine Techniques

### 9.1. What PET/CT Adds

^18^F-FDG PET/CT is best considered a complementary whole-body problem-solving tool rather than a replacement for MRI [32,33,84,85,86,87]. The 2020 meta-analysis by Treglia et al. reported pooled sensitivity and specificity of 94.8% and 91.4%, respectively, for spinal infection overall [84]. These data support PET/CT as a valuable modality in suspected spondylodiscitis, but not as an intrinsically pathogen-specific test [84,85,87]. The comparative advantages of PET/CT are now better defined. It is less affected by metallic hardware than MRI and can therefore be especially helpful in postoperative or implant-associated infection [85,86,88]. It also provides a wide field of view that may reveal metastatic infection, psoas abscesses or additional clinically silent vertebral foci beyond the symptomatic level [85,87,89]. PET/CT may also perform well early in the disease course, when marrow and disc changes are still subtle on anatomical imaging [84,90,91]. In the study by Smids et al., PET/CT had superior diagnostic value in the first two weeks of disease, whereas MRI and PET/CT performed similarly thereafter [91]. MRI nevertheless remains superior for small epidural abscesses and detailed assessment of canal compromise [89,91].

### 9.2. Aetiological Differentiation and Staging

The role of PET/CT in differentiating TS from PS is narrower than the role of MRI [92]. A small retrospective study by Bassetti et al. reported higher FDG uptake in TS than in bacterial spondylodiscitis, suggesting that SUVmax may occasionally increase suspicion for TB [92]. However, FDG uptake reflects inflammatory-cell metabolism rather than microbiological identity; therefore, neither SUVmax nor any isolated SUV threshold can reliably distinguish TS from PS [85,86,92]. PET/CT may be more useful in suspected TS for whole-body staging: the 2026 Spinal TB X cohort reported a high rate of disseminated disease on whole-body PET/CT, emphasizing that apparently focal spinal TB may represent part of a broader systemic process [93].

### 9.3. Limits of Interpretation

Interpretation nevertheless requires caution. Postoperative inflammation, active degenerative endplate changes, and some neoplasms may all produce false-positive FDG uptake, whereas recent antimicrobial exposure, low-grade infection and small-volume disease may reduce conspicuity [84,85,86,87,94]. PET/CT should therefore be interpreted alongside MRI, clinical context and microbiology when the question is specifically TS versus PS [84,86,92].

### 9.4. When PET/CT Is Most Useful

In practice, PET/CT is most helpful in four scenarios: contraindication to MRI, equivocal MRI, suspected multifocal or disseminated infection, and discordant follow-up in which symptoms or inflammatory markers improve but MRI remains difficult to interpret [84,86,87,94]. Recent reviews also describe potential roles in biopsy targeting and treatment-response assessment, although response thresholds are not yet standardized [86,95]. The registry analysis by Lang et al. linked PET/CT use with lower in-hospital mortality in pyogenic spondylodiscitis, especially in older adults, but that observation should be interpreted as associative and hypothesis-generating rather than causal [96]. If PET/CT is unavailable, SPECT/CT or other scintigraphic techniques may still identify infection, but they contribute less to etiological differentiation and are now best regarded as fallback rather than preferred options [7,11,97].

## 10. An Imaging-Led Diagnostic Pathway

**Figure 5:**
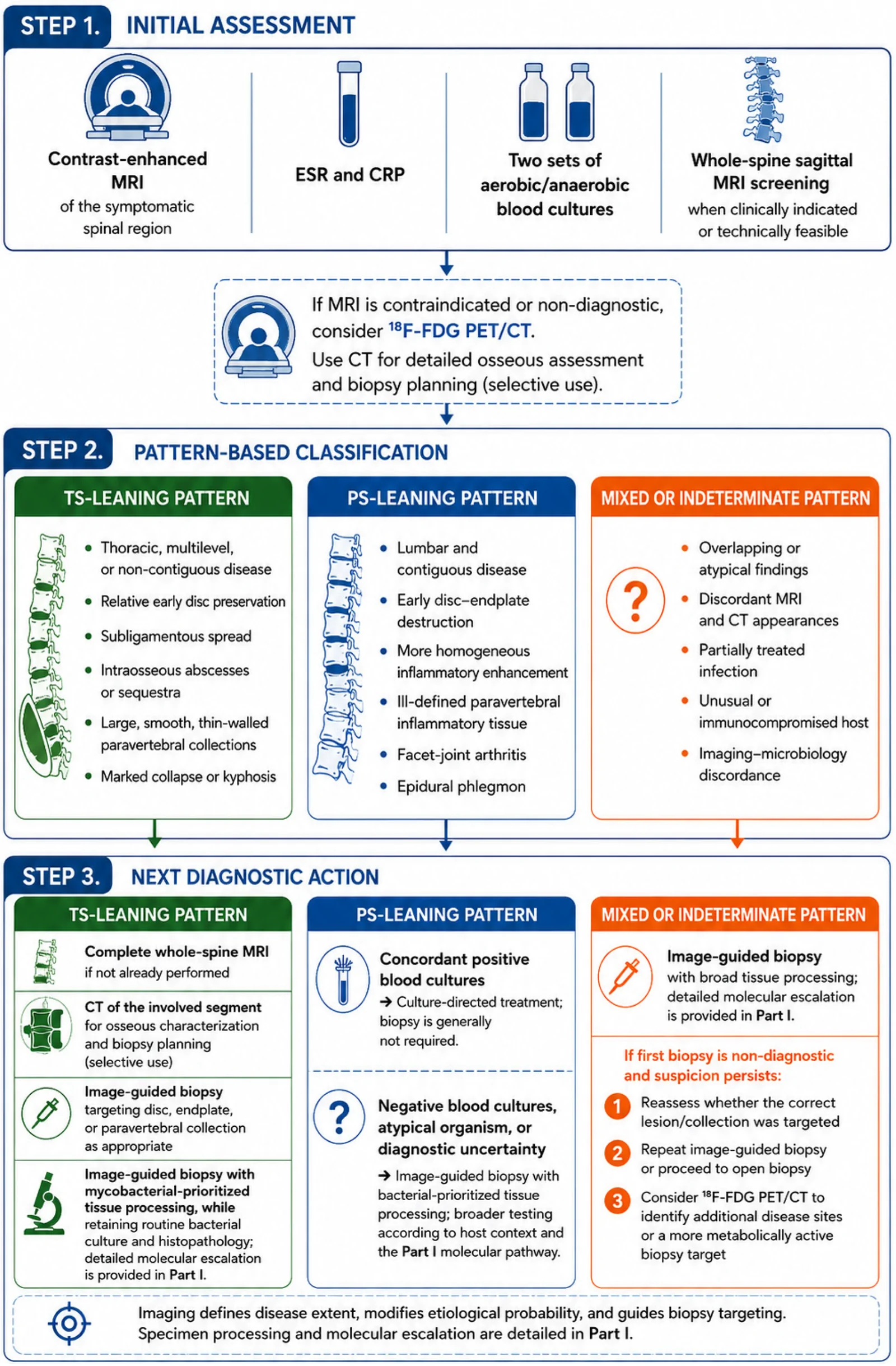
Practical imaging-led diagnostic algorithm for clinically stable patients with suspected native spondylodiscitis. Suggested sequencing of MRI, CT, PET/CT, and image-guided tissue sampling using pattern-based classification to guide whole-spine staging, biopsy targeting, and tissue-processing priorities in TS-leaning, PS-leaning, and mixed or indeterminate presentations. Imaging refines etiological probability and guides biopsy strategy but does not replace tissue confirmation; the detailed molecular escalation algorithm is presented in Part I. **Note**: Patients with neurological deficit, cord compression, sepsis, or hemodynamic instability require urgent surgical assessment and antimicrobial management, which should proceed in parallel with attempts to obtain microbiological and histopathological specimens whenever feasible. Follow-up is primarily clinical and biochemical; repeated MRI is reserved for neurological deterioration, poor clinical or inflammatory-marker response, or persistent diagnostic uncertainty.

In practice, an imaging-led pathway is most useful when it is sequential and biopsy-oriented. Contrast-enhanced regional MRI is usually the first test in suspected native spondylodiscitis [7,32,33]. When the pattern leans towards TS—thoracic disease, three or more segments, skip lesions, relative early disc preservation, subligamentous spread, intraosseous abscesses, large thin-walled well-defined paravertebral collections, marked collapse or kyphosis—it is reasonable to add whole-spine MRI and CT of the affected segment and to direct biopsy towards tissue suitable for mycobacterial microscopy, culture and nucleic-acid amplification [14,15,32,53]. When the pattern leans towards PS—lumbar disease, early disc-endplate destruction, relatively homogeneous inflammatory enhancement, facet-joint arthritis, and epidural phlegmon—standard bacterial microbiology should be prioritized, while still recognizing that atypical or partially treated PS may resemble TS [1,2,15,32]. PET/CT is best reserved for cases where MRI is contraindicated or equivocal, for postoperative hardware, or for suspected multifocal/disseminated disease [32,84,86]. When the phenotype is mixed, or the host context is unusual, the safer next step is broader tissue processing—including bacterial, mycobacterial, and fungal studies plus histopathology—rather than forcing a binary label [6,10,58]. Imaging should refine etiological probability and biopsy strategy, but should not replace tissue confirmation when tissue is feasible [6,7,94]. When MRI and CT findings are discordant, or when initial biopsy is non-diagnostic, broadening tissue processing (bacterial, mycobacterial, and fungal cultures plus histopathology and molecular testing) and considering PET/CT for whole-body staging or repeat image-guided biopsy from a PET-avid site is preferable to assigning a presumptive etiology on imaging pattern alone. A practical imaging-informed diagnostic pathway based on current evidence is summarised in Figure 5.

## 11. Limitations and Research Priorities

The imaging evidence base has expanded, but it remains heterogeneous. As a narrative review, this article aimed to synthesize clinically relevant evidence rather than to provide formal quality appraisal or pooled quantitative estimates. The structured yet iterative search and purposive, author-led evidence selection may have introduced selection and citation bias; broad database searching and hand-searching of reference lists were used to mitigate this risk but could not eliminate it. Publication and language bias are also possible because the search was restricted to English-language indexed literature. Much of the comparative evidence derives from retrospective, single-country or geographically localized cohorts, frequently from high-TB-burden referral settings and enriched for microbiologically confirmed cases. These features may exaggerate the contrast between TS and PS phenotypes encountered in routine mixed-probability practice and reduce the certainty and generalizability of both imaging-sign frequencies and prediction-model performance in lower-prevalence settings. Definitions of ‘epidural abscess’, ‘paravertebral abscess’, ‘skip lesion’, and ‘posterior element involvement’ also vary across studies, and MRI protocols are not standardized. Most prediction models have undergone only internal validation or limited external validation, and even strong recent data from 2024 to 2026 come predominantly from single-country cohorts. Emerging quantitative approaches, including EAS/VBS signal scores and radiomics, remain promising but are not yet broadly generalizable. Future work should prioritize independent multicenter validation, harmonized imaging definitions, calibration across low- and high-TB-prevalence settings, and closer integration of imaging with rapid mycobacterial molecular testing.

## 12. Conclusions

For clinicians, the most helpful imaging message is not that TS and PS have mutually exclusive appearances, but that they often occupy different ends of a spatial and biological spectrum. TS more often presents as a relatively slow, body-centered, subligamentous, and abscess-forming process, with disproportionate collapse and relative disc preservation early on; PS more often appears as an acute discovertebral and phlegmonous infection with earlier disc destruction and facet involvement. MRI remains the principal differentiating modality; CT adds structural detail and biopsy guidance; and PET/CT is most useful for selected problem-solving scenarios, whole-body staging, and detection of additional disease sites rather than for etiological separation, as SUV values alone cannot reliably distinguish TS from PS. Used together, these modalities can improve early etiological stratification and support more timely and better-targeted tissue sampling. However, the available evidence remains heterogeneous and is derived largely from retrospective, geographically localized cohorts, so imaging findings and prediction models should be interpreted cautiously. Ultimately, the role of imaging is to modify pre-test probability, narrow the etiological differential, and optimize tissue sampling; it should not substitute for microbiological or histological confirmation, which remains the diagnostic reference whenever feasible [10,58,77,94].

## Figures and Tables

**Table 3:** Features distinguishing infectious spondylodiscitis from a non-infectious mimic on MRI.

Findings Favoring Infection (PS or TS)	Findings Favoring a Non-Infectious Mimic *
Paraspinal or epidural inflammatory extension	Vacuum disc
Imaging severity concordant with the clinical/inflammatory phenotype	Absence of paraspinal or epidural inflammatory extension
Endplate edema and enhancement that progresses on interval imaging	Dominant posterior-element tumor mass
	Exuberant sclerosis or ossification
	Marked discordance between imaging severity and the inflammatory phenotype

* Representative mimic entities: Modic type 1 degeneration; acute Schmorl node; dialysis-related or neuropathic spondyloarthropathy; postoperative granulation tissue; SAPHO/Andersson lesions; malignant infiltration.

## Data Availability

No new data were created or analyzed in this study. Data sharing is not applicable to this article.

## Supplementary Materials

The following supporting information can be downloaded at https://www.mdpi.com/article/10.3390/diagnostics16162540/s1, Supplementary File S1: Full electronic search strategies used in this narrative review, including the complete Boolean search strings for PubMed/MEDLINE and Scopus with MeSH controlled vocabulary terms and free-text operators across three concept blocks; the list of five high-citation narrative reviews hand-searched for additional eligible records; and the pre-specified eligibility criteria and definitions of confirmed and probable spondylodiscitis applied during record screening.

## Abbreviations

The following abbreviations are used in this manuscript:

MRI	Magnetic resonance imaging
CT	Computed tomography
PET/CT	Positron emission tomography–computed tomography
TS	Tuberculous spondylodiscitis
PS	Pyogenic spondylodiscitis
^18^F-FDG	Fluorine-18 fluorodeoxyglucose
STIR	Short tau inversion recovery
SUV	Standardized uptake value
AUC	Area under the receiver operating characteristic curve
WBC	White blood cell count
ESR	Erythrocyte sedimentation rate
CE-MRI	Contrast-enhanced magnetic resonance imaging
AI	Artificial intelligence
TB	Tuberculosis
SAPHO	Synovitis, acne, pustulosis, hyperostosis, and osteitis
^18^F-FDG PET/CT	Fluorine-18 fluorodeoxyglucose positron emission tomography–computed tomography
SUVmax	Maximum standardized uptake value
SPECT/CT	Single-photon emission computed tomography/computed tomography
DCE-MRI	Dynamic contrast-enhanced magnetic resonance imaging
EAS	Epidural abscess signal
VBS	Vertebral body signal
