# Supplementary material for: Differentiating Tuberculous and Pyogenic Spondylodiscitis: Part II: MRI/CT Features, Prediction Models, and the Role of PET/CT—A Narrative Review
Source: Diagnostics (Basel). 2026 Aug 12;16(16):2540. doi: 10.3390/diagnostics16162540 (PMC13511338; doi:10.3390/diagnostics16162540)
Supplement: Supplementary file 1 [file diagnostics-16-02540-s001.zip › diagnostics-4379571-supplementary.pdf]

Supplementary File S1

Full Electronic Search Strategies and Eligibility Framework

Differentiating Tuberculous and Pyogenic Spondylodiscitis: Part II. MRI/CT Features, Prediction Models, and the Role of PET/CT

Purpose. This Supplementary File supports the revised Methods by reporting the literature sources, search limits, search architecture, controlled-vocabulary mapping, complete database-specific Boolean strings, reference-list screening, eligibility criteria, operational definitions, and reference-category summary used for the narrative review.

Abbreviations: CT, computed tomography; FDG, fluorodeoxyglucose; MeSH, Medical Subject Headings; MRI, magnetic resonance imaging; PET/CT, positron emission tomography/computed tomography; PS, pyogenic spondylodiscitis; SPECT/CT, single-photon emission computed tomography/computed tomography; TS, tuberculous spondylodiscitis.

S1.1 Literature sources and coverage

Database	Interface / platform	Coverage searched	Role
PubMed/MEDLINE	PubMed.gov (NLM)	Database inception to 31 March 2026	Primary electronic search
Scopus	Scopus.com (Elsevier)	Database inception to 31 March 2026	Secondary electronic search using translated syntax; no additional eligible records identified

S1.2 Search limits

Parameter	Setting
Date range	Database inception – 31 March 2026
Language	English
Species	Humans
Publication type at search stage	No restriction by article type. Original research, meta-analyses, and pictorial/narrative reviews with primary imaging data were eligible for screening. Conference abstracts without full text were excluded at full-text screening, not at the search-filter stage.
Age group filter	None applied at the search stage. Pediatric records were retained for screening; pediatric-only studies were subsequently handled as described in the Methods section.

S1.3 Search architecture and concept blocks

The electronic strategy combined three concept blocks using the logic: Block 1 AND (Block 2 OR Block 3). Each record was required to include a condition term combined with either an imaging-modality term or an evidence-type term. Terms within each block were combined with OR.

Block	Concept	Terms combined with OR within the block
1	Condition	tuberculous spondylitis; tuberculous spondylodiscitis; spinal tuberculosis; pyogenic spondylodiscitis; pyogenic spondylitis; vertebral osteomyelitis; spondylodiscitis
2	Imaging modality	MRI; magnetic resonance imaging; computed tomography; PET/CT; FDG; 18F-FDG; whole-spine MRI; nuclear medicine; SPECT/CT
3	Evidence type / imaging descriptor	prediction model; radiomics; deep learning; diagnostic accuracy; imaging features; abscess; sequestra; subligamentous spread

S1.4 Controlled-vocabulary and free-text mapping

Where an appropriate MeSH heading existed, the heading was combined with the corresponding title/abstract free-text term to maximize sensitivity. Terms without a dedicated or consistently indexed MeSH heading were searched as free-text only.

Block	Free-text term	Controlled-vocabulary term / MeSH heading	Mapping
1	tuberculous spondylitis / spinal tuberculosis	Tuberculosis, Spine	Yes
1	pyogenic spondylodiscitis / pyogenic spondylitis	—	No dedicated heading; free text only
1	vertebral osteomyelitis	Osteomyelitis combined with Spine[tiab]	Partial
1	spondylodiscitis	Discitis	Yes; closest indexed heading

Block	Free-text term	Controlled-vocabulary term / MeSH heading	Mapping
2	MRI / magnetic resonance imaging	Magnetic Resonance Imaging	Yes
2	computed tomography	Tomography, X-Ray Computed	Yes
2	PET/CT	Positron Emission Tomography Computed Tomography	Yes
2	FDG / 18F-FDG	Fluorodeoxyglucose F18	Yes
2	nuclear medicine / SPECT/CT	Tomography, Emission-Computed, Single-Photon	Partial
3	prediction model / diagnostic accuracy	Diagnostic Techniques and Procedures subheadings	Partial
3	radiomics	—	No dedicated heading; indexed only since approximately 2021 and inconsistently; free text only
3	deep learning	Deep Learning	Yes
3	abscess / sequestra / subligamentous spread	Abscess	Partial; abscess only. Sequestra and subligamentous spread were searched as free text only

S1.5 Complete PubMed/MEDLINE search strategy

Search fields combined title/abstract [tiab] terms with exploded MeSH headings [Mesh], using the logic specified in S1.3.

```
("tuberculous spondylitis"[tiab] OR "tuberculous spondylodiscitis"[tiab] OR "Tuberculosis, Spine"[Mesh] OR "spinal tuberculosis"[tiab] OR "pyogenic spondylodiscitis"[tiab] OR "pyogenic spondylitis"[tiab] OR "vertebral osteomyelitis"[tiab] OR "spondylodiscitis"[tiab] OR "Discitis"[Mesh])
AND
(("MRI"[tiab] OR "magnetic resonance imaging"[tiab] OR "Magnetic Resonance Imaging"[Mesh] OR "computed tomography"[tiab] OR "Tomography, X-Ray Computed"[Mesh] OR "PET/CT"[tiab] OR "Positron Emission Tomography Computed Tomography"[Mesh] OR "FDG"[tiab] OR "18F-FDG"[tiab] OR "Fluorodeoxyglucose F18"[Mesh] OR "whole-spine MRI"[tiab] OR "nuclear medicine"[tiab] OR "SPECT/CT"[tiab])
OR
("prediction model"[tiab] OR "radiomics"[tiab] OR "deep learning"[tiab] OR "Deep Learning"[Mesh] OR "diagnostic accuracy"[tiab] OR "imaging features"[tiab] OR "abscess"[tiab] OR "sequestra"[tiab] OR "subligamentous spread"[tiab]))
Filters/limits: Humans; English.
Date range: database inception – 31 March 2026.
```

S1.6 Complete Scopus search strategy

The same concept blocks and date range were translated into Scopus syntax using TITLE-ABS-KEY() field searching. This secondary search identified no additional eligible records beyond those already retrieved from PubMed/MEDLINE.

```
TITLE-ABS-KEY(
  ("tuberculous spondylitis" OR "tuberculous spondylodiscitis" OR "spinal tuberculosis" OR "pyogenic spondylodiscitis" OR "pyogenic spondylitis" OR "vertebral osteomyelitis" OR "spondylodiscitis")
  AND
  (("MRI" OR "magnetic resonance imaging" OR "computed tomography" OR "PET/CT" OR "FDG" OR "18F-FDG" OR "whole-spine MRI" OR "nuclear medicine" OR "SPECT/CT")
  OR
  ("prediction model" OR "radiomics" OR "deep learning" OR "diagnostic accuracy" OR "imaging features" OR "abscess" OR "sequestra" OR "subligamentous spread"))
)
AND PUBYEAR < 2027
AND (LIMIT-TO(LANGUAGE, "English"))
```

S1.7 Manual reference-list screening

Reference lists of the following five high-citation imaging-focused narrative reviews published after 2015 were manually screened by two co-authors to identify additional eligible records not retrieved by the electronic database searches:

1. Crombé, A. et al. Imaging of spondylodiscitis: A comprehensive updated review — Multimodality imaging findings, differential diagnosis, and specific microorganisms detection. Microorganisms 2024, 12, 893. [ref. 7].
2. Laur, O. et al. Imaging assessment of spine infection. Skeletal Radiol. 2024, 53, 2067–2079. [ref. 8].
3. Raghavan, M.; Palestro, C.J. Imaging of spondylodiscitis: An update. Semin. Nucl. Med. 2023, 53, 152–166. [ref. 9].

4. Hirunpat, P. et al. Imaging clues for the diagnosis of various pathogenic causes of infectious spondylitis. Skeletal Radiol. 2025, 54, 2411–2424. [ref. 10].
5. Dhodapkar, M.M. et al. Imaging in spinal infections: Current status and future directions. N. Am. Spine Soc. J. 2023, 16, 100275. [ref. 11].

These reviews were selected as the broadest and most recent non-comparative imaging overviews in the reference list. Comparative TS-versus-PS primary studies were identified directly through the database search strings.

S1.8 Eligibility criteria and operational definitions

Records were eligible if they reported MRI, CT, or PET/CT findings in patients with confirmed or probable spondylodiscitis, or if they directly compared imaging features, diagnostic scores, or nuclear medicine findings between TS and PS on at least one parameter.

Domain	Definition / criterion
Confirmed spondylodiscitis	Microbiologically established disease by a positive culture or a validated molecular test from spinal or paraspinal tissue or blood.
Probable spondylodiscitis	Clinical and radiological diagnosis meeting Infectious Diseases Society of America (IDSA) 2015 or Société de Pathologie Infectieuse de Langue Française (SPILF) 2022 guideline criteria in the absence of microbiological confirmation.
Prediction-model and radiomics studies	Included only if internal or external validation performance was reported.
Postoperative, hardware-associated, or pediatric-only imaging studies	Used to inform corresponding dedicated subsections rather than the main comparative synthesis.
Case reports and small case series	Series of fewer than 10 patients were excluded unless they documented imaging findings not available in larger series.

S1.9 Reference-set category summary

The final narrative reference set comprised 97 records. The table below groups records according to their primary role in the narrative synthesis; categories are descriptive and are not intended as a PRISMA flow count.

Primary reference category	Count
Reviews, guidelines, and general background/epidemiology sources	27
Comparative TS-versus-PS MRI/CT imaging studies	24
PET/CT and nuclear medicine studies	14
Prediction-model, radiomics, and deep-learning studies	11
CT-specific studies	7
Pediatric spinal infection studies	6
Mimics and differential-diagnosis sources	6
Whole-spine imaging studies	2
Total	97
